# Elucidating the molecular responses to waterlogging stress in onion (*Allium cepa* L.) leaf by comparative transcriptome profiling

**DOI:** 10.3389/fpls.2023.1150909

**Published:** 2023-08-08

**Authors:** Pranjali A. Gedam, Kiran Khandagale, Dhananjay Shirsat, A. Thangasamy, Onkar Kulkarni, Abhijeet Kulkarni, Swaranjali S. Patil, Vitthal T. Barvkar, Vijay Mahajan, Amar Jeet Gupta, Kiran P. Bhagat, Yogesh P. Khade, Major Singh, Suresh Gawande

**Affiliations:** ^1^ Indian Council of Agricultural Research (ICAR)-Directorate of Onion and Garlic Research, Pune, India; ^2^ Bioinformatics Centre, Savitribai Phule Pune University, Pune, India; ^3^ Department of Botany, Savitribai Phule Pune University, Pune, India; ^4^ Indian Council of Agricultural Research (ICAR)-Directorate of Floriculture Research, Pune, India

**Keywords:** waterlogging, onion, transcriptome, RNA-sequencing, differential gene expression

## Abstract

**Introduction:**

Waterlogging is a major stress that severely affects onion cultivation worldwide, and developing stress-tolerant varieties could be a valuable measure for overcoming its adverse effects. Gathering information regarding the molecular mechanisms and gene expression patterns of waterlogging-tolerant and sensitive genotypes is an effective method for improving stress tolerance in onions. To date, the waterlogging tolerance-governing molecular mechanism in onions is unknown.

**Methods:**

This study identified the differentially expressed genes (DEGs) through transcriptome analysis in leaf tissue of two onion genotypes (Acc. 1666; tolerant and W-344; sensitive) presenting contrasting responses to waterlogging stress.

**Results:**

Differential gene expression analysis revealed that in Acc. 1666, 1629 and 3271 genes were upregulated and downregulated, respectively. In W-344, 2134 and 1909 genes were upregulated and downregulated, respectively, under waterlogging stress. The proteins coded by these DEGs regulate several key biological processes to overcome waterlogging stress such as phytohormone production, antioxidant enzymes, programmed cell death, and energy production. The clusters of orthologous group pathway analysis revealed that DEGs contributed to the post-translational modification, energy production, and carbohydrate metabolism-related pathways under waterlogging stress. The enzyme assay demonstrated higher activity of antioxidant enzymes in Acc. 1666 than in W-344. The differential expression of waterlogging tolerance related genes, such as those related to antioxidant enzymes, phytohormone biosynthesis, carbohydrate metabolism, and transcriptional factors, suggested that significant fine reprogramming of gene expression occurs in response to waterlogging stress in onion. A few genes such as ADH, PDC, PEP carboxylase, WRKY22, and Respiratory burst oxidase D were exclusively upregulated in Acc. 1666.

**Discussion:**

The molecular information about DEGs identified in the present study would be valuable for improving stress tolerance and for developing waterlogging tolerant onion varieties.

## Introduction

Water is a prerequisite for plant growth; however, excess soil moisture or prolonged soil saturation causes waterlogging stress that adversely affects several plant developmental processes. Every year, approximately 17 million km^2^ of land area and 16% of global agriculture production are challenged by flooding stress (Kaur et al., 2020). The reasons for waterlogging in the field is soil type, poor soil drainage, and unpredictable and intense rainfall. Waterlogging affects plant vegetative growth as well as strongly influences its reproductive growth, leading to yield loss and sometimes complete crop failure. In the context of global climate change, the area under waterlogging stress has been increasing every year since the 1990s, affecting 10%–16% of soils and approximately 80% of the yield losses of economically important crops (Shabala, 2011).

Onion (*Allium cepa* L.) is a significantly valuable crop cultivated globally on approximately 7.1 million-hectare land area. The annual bulb production is 136 million tonnes (FAO, 2021). Next to China, India is the second largest onion producer contributing to 22% of global production (Priyadarshini et al., 2021). Depending on the amount and distribution of rainfall, onion in India is cultivated as both a rainfed and irrigated crop during the monsoon and post-monsoon seasons, respectively. Waterlogging or soil flooding due to heavy rainfall occurs in a substantial part of the world. In India, the monsoon is the major irrigation water source for *Kharif* onion crops (July–December). Erratic monsoon rains lead to frequent waterlogging episodes. Onion is extremely sensitive to waterlogging stress as it is a shallow-rooted crop (Pelter et al., 2004). Heavy and prolonged rainfall at almost all growth stages restrains crop growth with 50%–70% yield losses, indicating that the onion crop is sensitive to waterlogging (Ghodke et al., 2018). Thus, waterlogging is a grave environmental threat limiting onion production during monsoons. To attain adequate onion production during monsoons, genotypes that can withstand short or prolonged waterlogging conditions must be identified. In our previous study, we screened diverse onion genotypes for waterlogging tolerance and then grouped them as tolerant and sensitive based on their phenotypic and physiological traits (Gedam et al., 2022).

In waterlogged soil, the main reason for plant growth inhibition and mortality is a low oxygen diffusion rate that creates a hypoxic condition in roots, thereby shifting the energy-related aerobic respiration pathway to the anaerobic fermentation mode and declining plant root activity, permeability, and growth (Zhou et al., 2020). This disturbs a series of morphological, physiological, and biochemical mechanisms, thereby limiting plant development, gas exchange, and photosynthesis-related processes. Waterlogging triggers the production of reactive oxygen species (ROS) that are detrimental to the cellular membrane components. They also inactivate various enzyme activities, cause oxidation of proteins and membrane lipids, and damage nucleoproteins (Yeung et al., 2018).

Plants respond to waterlogging stress by undergoing a series of phenotypic, physiological, biochemical, and molecular alterations. Changes in leaf and root morphological and anatomical structures are the first visual morphological adaptations that occur in response to stress. Likewise, physiological and molecular regulatory mechanisms, such as reduction in stomatal conductance, a decline in photosynthesis, activation of certain hormonal biosynthesis and signaling pathways, induction of antioxidant enzyme systems, and modulation of certain genes and transcription factors, are triggered in response to waterlogging stress (Pan et al., 2021; Wu et al., 2022). Plant hormonal regulation is a crucial player in plant development and stress response. Ethylene is a key hormone regulating the waterlogging stress response in plants. It is synthesized by 1-aminocyclopropane-1-carboxylic acid (ACC) synthase and ACC oxidase. Ethylene and ROS signaling leads to programmed cell death (PCD) in root tissues, thereby resulting in aerenchyma formation that mediates gaseous exchange during waterlogging (Khan et al., 2020). Salicylic acid (SA) is another phytohormone involved in activating antioxidants and aerenchyma formation (Koramutla et al., 2022) during waterlogging and hypoxia conditions. Energy crisis is common under waterlogging stress and is caused by limited oxygen supply. Therefore, anaerobic fermentation is initiated for maintaining ATP production through glycolysis by oxidation of NADH to NAD^+^. The higher expression of *pyruvate decarboxylase (PDC)* and *alcohol dehydrogenase (ADH)* was reported in waterlogging-tolerant genotypes, which helps to meet the temporary demand of ATP with alcohol fermentation (Xu et al., 2014; Luo et al., 2017; Zhang et al., 2017; Shen et al., 2021). Further, overexpression of these genes also reported to be associated with better performance of transgenic plants under waterlogging conditions than wild-type plants (Komatsu et al., 2011; Tougou et al., 2012; Zhang et al., 2016). Transcription factors are a critical player in regulating gene expression in response to stress. Gene regulation under the waterlogging or hypoxic condition depends on group VII *Ethylene Response Factors* (*ERFs*). They act as master inducers of several hypoxia-responsive genes (Gibbs et al., 2011; Gasch et al., 2016). The aforementioned adaptive processes facilitate acclimatization of the plant to the detrimental consequences of waterlogging stress.

RNA-Seq technology is a powerful and cost-effective tool for conducting the high-throughput plant transcriptome analysis and thus identifying candidate genes associated with various stress-responsive metabolic pathways. This technology has been efficiently used for understanding the expression pattern of key genes and transcription factors associated with waterlogging stress-responsive metabolic pathways in crops such as potatoes and cassava (Park et al., 2020; Cao et al., 2022). Although roots are the key part affected by waterlogging, researchers have also investigated the waterlogging stress response in the leaf tissues of soybean (Chen et al., 2016), peanut (Zeng et al., 2021), and pigeon pea (Tyagi et al., 2022).

To date, no study has highlighted the molecular mechanism governing waterlogging stress tolerance in onion crops. The present study compared the waterlogging-tolerant onion genotype with the sensitive one at the transcriptional level to identify waterlogging tolerance-associated key genes and related pathways. Both genotypes were selected based on previous findings of phenotypic and biochemical responses under the waterlogging condition (Gedam et al., 2022). To the best of our knowledge, this study is the first to investigate the molecular mechanism underlying waterlogging tolerance in onion.

## Material and methods

### Plant material

Two onion genotypes (*Allium cepa* L.) with contrasting responses to waterlogging stress were used. The genotype Acc. 1666 exhibited a high tolerance to waterlogging stress, whereas W-344 was highly sensitive (Gedam et al., 2022). The experiment was conducted at the ICAR-Directorate of Onion and Garlic Research, Pune, India (N 18˚84′, E 73˚88′, H 553.8 m). Seedlings of both genotypes were raised in plastic pots (100 × 150 × 60 cm, height × length × width) filled with a 1:3 ratio of farm yard manure and clay loam soil. The recommended fertilizer and plant protection measures were followed to grow healthy seedlings. The experiment was conducted in a completely randomized block design with 10 replications per treatment for each genotype. The seedlings were raised under ambient growth conditions and irrigated after every 36 h until the 5–6 leaf stage.

### Waterlogging treatment

After 45 days of seedling transplantation, half of the pots for each genotype were placed in a tank where the water level was maintained 4 cm above the soil surface to induce waterlogging stress (**Figure S1**). Each pot contained 10 seedlings, and the waterlogged condition was maintained for 72 h. The remaining half pots were maintained in ambient growth conditions throughout the experiment under a rain-out shelter. The leaf samples were collected from both control and waterlogged plants before the stress was relieved, immediately frozen in liquid nitrogen, and stored at −80°C.

### Morphological and physiological traits

The phenotypic traits, namely plant height, leaf length, and the number of leaves per plant, were recorded 72 h after the stress treatment (control and waterlogging) in five replications from both genotypes. The fourth fully expanded leaf was cut, and leaf area was measured using the leaf area meter. The seedlings from both control and waterlogged pots were carefully uprooted and washed after the stress period, and their shoot and root lengths were recorded. Leaf total chlorophyll content was quantified using the method of Hiscox and Israelstam (1979). Leaf tissue (0.05 g) was weighed and placed in a test tube containing 10 mL dimethyl sulfoxide. The test tubes were incubated at 60°C for 1 h in water bath and allowed to cool at room temperature for 30 min. Absorbance was recorded at 645 and 663 nm by using a UV–visible spectrophotometer (Thermo Fisher Scientific, Waltham, MA, USA). The total chlorophyll content was calculated using the following formula given by Arnon (1949):


$$\text{Chlorophyll content}=\frac{(20.2\times \text{OD}645+8.02\times \text{OD}663)\times \text{Volume of the extract}}{1000\times \text{Weight of the sample}}$$

where OD_663_ and OD_645_ are absorbances at 663 and 645 nm, respectively.

Lipid peroxidation in onion leaves was estimated using the protocol of Heath and Packer (1968). The lipid peroxidation level was measured in terms of thiobarbituric acid reactive substance (TBARS) contents by reading absorbance at 532 nm by using a spectrophotometer. The TBARS content level is expressed as μmol g^−1^ FW. The hydrogen peroxide level was quantified using the method of Rao et al. (1997). The onion leaf sample (1 g) was pulverized using liquid nitrogen, followed by the addition of 10 mL ice-cooled acetone. The homogenate was filtered, and 4 mL of titanium reagent (This reagent was prepared according to the method of Teranishi et al., 1974); then, 5 mL of ammonium solution was added to form a titanium hydroperoxide complex. The mixture was centrifuged at 10000 g for 10 min, and the precipitate obtained was dissolved in 10 mL of 2 M sulphuric acid and recentrifuged. The absorbance of the supernatant was read at 415 nm by using the UV–Visible spectrophotometer. The H_2_O_2_ concentration was computed by referring to the standard curve and is expressed as μmol H_2_O_2_ g^−1^ FW.

### Antioxidant enzyme activity

Leaf samples were harvested at 72 h of waterlogging from both control and stressed plants of both genotypes. Catalase (CAT), ascorbate peroxidase (APX), superoxide dismutase (SOD), and peroxidase (POD) were assayed. Enzyme extraction was performed by grinding the leaf tissue in liquid nitrogen and extracting it in phosphate buffer (0.1 M) of pH 7.2, as described by Roylawar and Kamble (2017). Catalase activity was measured by recording the change in absorbance at 240 nm (Volk and Feierabend, 1989) due to H_2_O_2_ decomposition. For CAT activity estimation, 3 mL of the reaction mixture was formed by adding 0.1 M potassium phosphate buffer (pH 7.0), 30 mM H_2_O_2_, and distilled water to the enzyme extract. One unit of CAT activity was recorded as the amount of enzyme that used 1 mmol H_2_O_2_ per minute. SOD activity was determined according to the method of Beauchamp and Fridovich (1971). SOD activity was measured by adding the enzyme extract to a reaction mixture containing phosphate buffer (50 mM, pH 7.8), riboflavin (60 mM), methionine (20 mM), EDTA (1 mM), nitro-blue tetrazolium (1 mM), and distilled water. The absorbance was recorded at 560 nm after illuminating the sample under light for 20 min. One unit of SOD activity was considered as the amount of enzyme that caused 50% inhibition of NBT reduction in light compared with that in tubes without the enzyme. APX activity was determined by adding the enzyme extract to the reaction mixture containing Tris-HCL buffer (5 M, pH 7.6), Na_2_EDTA (0.1 mM), ascorbic acid (0.5 mM) and distilled water. The reaction was initiated by adding H_2_O_2_, and the activity was measured by recording the reduction in ascorbate at 290 nm, as described by Nakano and Asada, 1987. POD activity was estimated according to the method of Castillo et al. (1984), wherein the enzyme extract was added to the reaction mixture comprising phosphate buffer (50 mM, pH 6.1), guaiacol (16 mM), H_2_O_2_ (2 mM), and distilled water. The POD activity involved the oxidation of guaiacol to tetra-guaiacol and was determined by reading the absorbance at 470 nm.

### RNA isolation and library preparation

Total RNA was isolated from the leaf tissues of control and waterlogged plants of both genotypes by using the RNeasy Plant Mini Kit (Qiagen, Germany). For each treatment, RNA was isolated from the leaf samples of three plants that were considered as one replicate. Three such independent biological replicates were used in the present study. Total RNA from each replicate was quantified and qualified using NanoDrop 1000 (Thermo Fisher Scientific, Waltham, MA, USA) and through 1% agarose gel analysis. Further, the RIN value of the isolated RNA was determined using Agilent 2100 Bioanalyzer (Agilent Technologies, Palo Alto, CA, USA). An equal amount of high-quality RNA from each replicate of the control and stressed samples of each genotype was used for library preparation. In total, 12 next-generation sequencing libraries were prepared using the NEBNext^®^Ultra™ RNA Library Preparation Kit for Illumina^®^ (NEB, Ipswich, MA, USA) as per the manufacturer’s protocol. The libraries were then sequenced in the paired-end mode with 150 × 2 read length by using the Illumina Hi-Seq 2500 platform.

### Transcriptome data analysis

The raw reads were filtered using the FastQC tool v0.12.1 with Q > 30, and only qualifying paired-end reads were considered for the analysis. The onion genome sequence v. 1.2 was downloaded from https://www.oniongenome.wur.nl/ with annotation files. The genome index was built using the STAR aligner with runModegenomeGenerate. The filtered high-quality paired reads were aligned to the reference genome and quantified using STAR aligner v. 2.7.10 (https://github.com/alexdobin/STAR). DESeq2 in R package was used for differential gene expression profiling between the control and treated samples of both genotypes. Differentially expressed transcripts were selected based on the following cut-offs: log2 fold change = 2 and p = 0.05. In addition to the information from the annotation file, the transcriptome was annotated using the DIAMOND BLASTX v. 2.0.9.147 (https://ab.inf.uni-tuebingen.de/software/diamond) tool against the UniProt/SwissProt database (https://www.uniprot.org/) and plant TFDB (http://planttfdb.cbi.pku.edu.cn/), with an e-value cut-off of ≤10^−2^. UniProt/SwissProt ID mapping functionality was used to obtain gene ontology (GO) and pathway annotation for the transcripts.

### Validation of DEGs under waterlogging stress through quantitative real-time PCR analysis

The expression of the randomly selected differentially expressed transcripts encoding waterlogging stress-responsive genes was validated through qRT-PCR (**Table 1**). RNA was isolated from the same samples that were employed for the transcriptome analysis. For each sample, three replicates were used in the study. RNA was treated with *DNase-I* (Fermentas, Lithuania) to eliminate DNA contamination. First-strand cDNA was synthesized from 1 µg RNA by using the RevertAid First Strand cDNA synthesis kit (Fermentas, Lithuania) following the manufacturer’s protocol. Primer-BLAST (https://www.ncbi.nlm.nih.gov/tools/primer-blast/) was used to design primers for selected genes where *AcActin* was used as a reference gene. The expression of selected genes was analyzed in the Realflex2 Master Cycler (Eppendorf, Germany). The reaction was carried out in 10 μL mixture containing 1× SYBR Green I master mix (Roche, Germany), 1 μL cDNA, and 1 µM of each primer. The relative gene expression and fold change were calculated using the 2^–ΔΔCT^ method (Livak and Schmittgen, 2001).

**Table 1:** Details of primers used for the validation of RNAseq data.

Transcript ID	Transcript Name	Primer sequence (5’-3’)	Product size (bp)
g107775	ACC Oxidase	GATCCTGCCCTGTTGACTTT	175
		CCAACACCCTATGCTTCACA	
g346683	Lipoxygenase	CCATGTATGCTCGTCCTGTC	163
		AGATGTCCAAATCGCTCGTC	
g165028	Alcohol dehydrogenase	GCCGGTCAGGTTATCAAGTG	134
		CTGACCCTTAGCTTCCCAGA	
g325946	Pyruvate decarboxylase	ATGTTGTCGGGTGATACTGC	199
		AGCTTCCATCACCAATGCAA	
g125872	WRKY70	ACACCGTCCAAAGAGAACAC	120
		CTCCCAATCTGTCATCCACC	
g238720	MYB1R1	TGGAAAAGGAGATTGGCGAG	156
		TCAGCAGTCGTTCCAACATC	
g444194	Respiratory burst oxidase	AGCGGTTCGTGAGAAAAAGT	142
		GATAGCAGGGCATTTGACGA	
Housekeeping gene	*AcActin*	GCACCAAGAGCAGTATTC	183
		CCAAATCTTCTCCATGTCA	

### Statistical analysis

One-way analysis of variance was performed to analyze data by using SAS software (version 9.3; SAS Institute, Cary, NC, USA). The results are presented as the mean ± standard error. The differences in the mean values were compared using Duncan’s multiple range test where a *p* value of<0.05 represented statistical significance.

## Results

### Physiological and biochemical parameters under waterlogging stress

**Figure 1:**
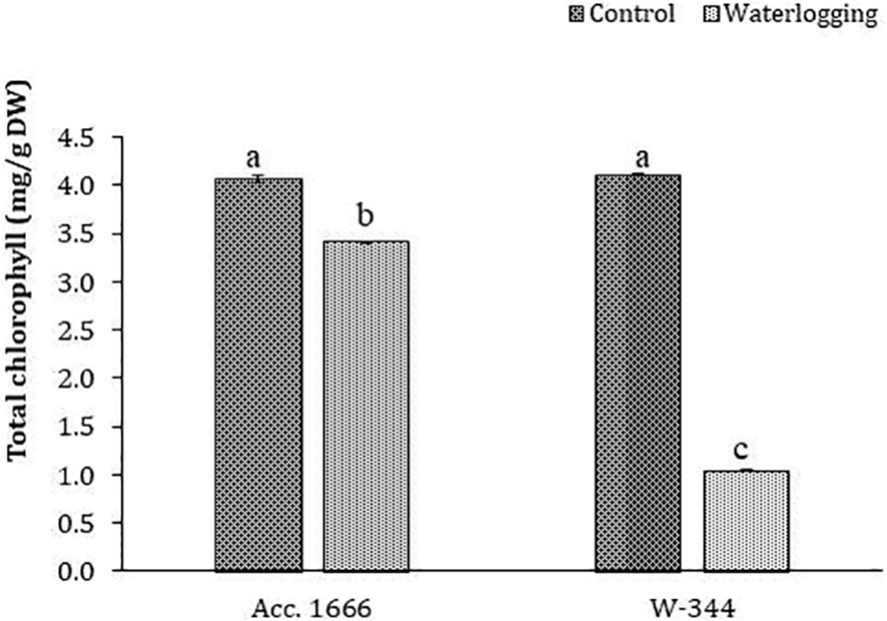
Chlorophyll content in contrasting onion genotypes after waterlogging stress. The different letters indicate statistically significant differences at *p*< 0.05.

**Figure 2:**
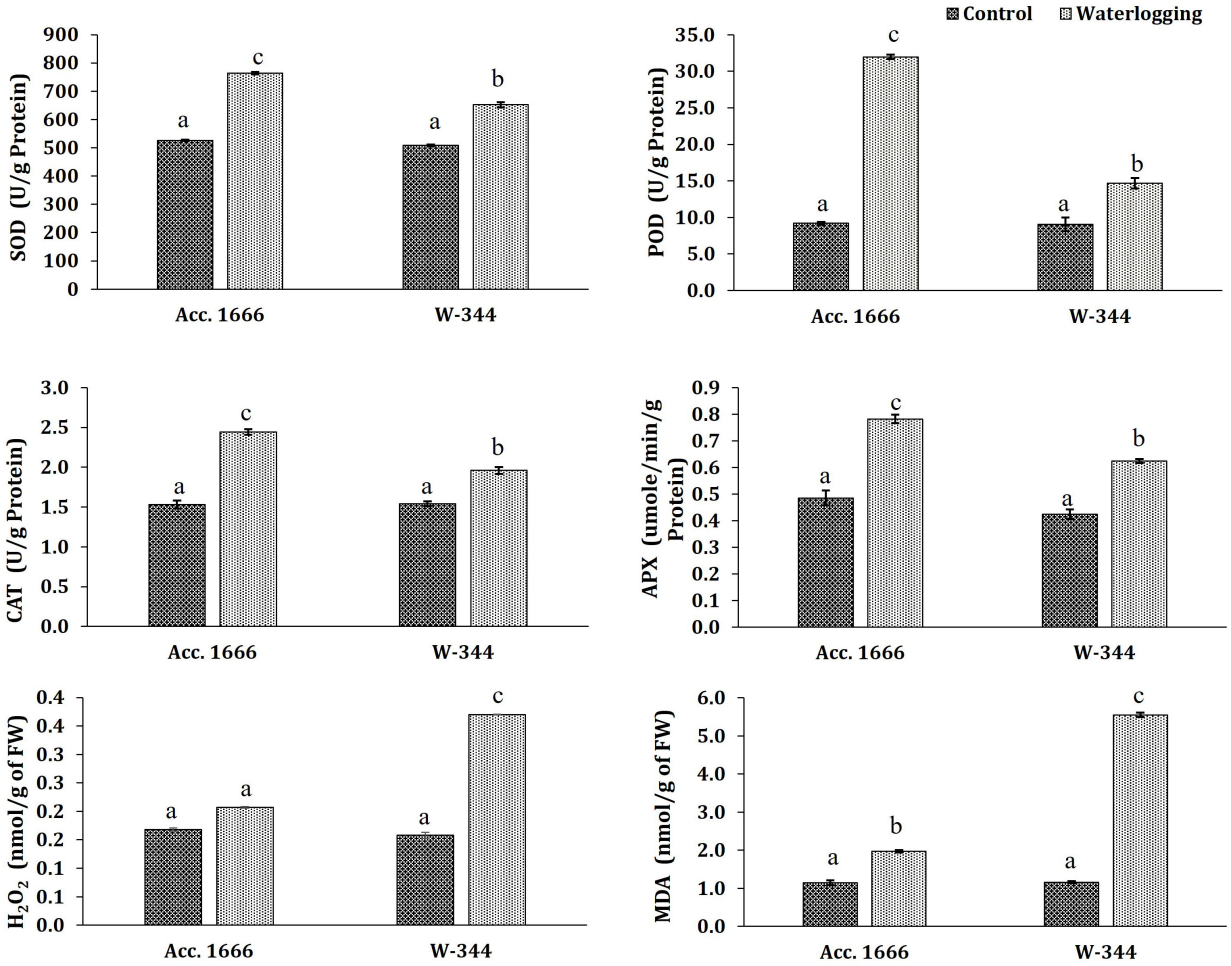
Effects of waterlogging stress on antioxidant enzyme activity in contrasting onion genotypes under waterlogging stress. Values are expressed as the average of three replicates, each consisting of three plants pooled together. The different letters indicate statistically significant differences at *p*< 0.05).

To understand the waterlogging tolerance mechanism in onion, physiological and biochemical analyses were conducted using waterlogging-tolerant (Acc. 1666) and sensitive (W-344) genotypes. Phenotypic traits such as plant height, leaf length, number of photosynthetically active leaves, and leaf area in the tolerant genotype were significantly better than those in the sensitive genotype under both water regimes (**Figure S2**). The aforementioned traits markedly declined in W-344 during stress, reflecting its poor growth performance under waterlogging stress. However, Acc. 1666 could maintain its plant height and photosynthetically active leaves to continue its growth in a stressful environment. Acc. 1666 maintained its chlorophyll level, whereas W-344 exhibited a significant reduction in the chlorophyll level under waterlogging stress (**Figure 1**). The H_2_O_2_ level was elevated in the sensitive genotype (W-344) but not in the tolerant genotype, under waterlogging stress (**Figure 2**). Further, antioxidant enzyme activities were induced due to waterlogging in both genotypes. Significantly higher levels of SOD, CAT, POD, and APX activities were observed in Acc. 1666 than in W-344 under waterlogging (**Figure 2**). This increase in antioxidant enzyme activity facilitated the tolerant genotype Acc. 1666 to withstand waterlogging with minimum growth damage.

### RNA-seq and *de novo* transcriptome assembly

Paired-end RNA sequencing of control and waterlogged samples of Acc.1666 and W-344 genotypes yielded 41194433 to 51240147 raw reads, respectively. The quality check analysis yielded 79%–85% high-quality reads for use in further analysis. The overall alignment rate of these reads with the onion reference genome was 84%–90% (**Table 2**). The raw RNA sequencing data were submitted to the NCBI (BioProject: PRJNA926808).

**Table 2:** Summary of RNA-seq data and its alignment with reference onion genome.

Control/Treatment	File Name	Bio Replicate	Total reads	Total HQ reads	Percentage HQ reads	Overall alignment with Onion ref genome
**Control**	1666_C1	BioRep1	93859624	76782844	81.81%	87.14%
	1666_C2	BioRep2	82388866	67921678	82.44%	87.37%
	1666_C3	BioRep3	76703980	60804856	79.27%	87.00%
**Waterlogging**	1666_WL	BioRep1	96634572	77980830	80.70%	88.65%
	1666_WL2	BioRep2	90456116	72938486	80.63%	89.21%
	1666_WL3	BioRep3	102480294	91617856	89.40%	89.71%
**Control**	344_C1	BioRep1	98707754	80641202	81.70%	90.07%
	344_C2	BioRep2	91684122	75300356	82.13%	90.05%
	344_C3	BioRep3	92127778	76191504	82.70%	89.91%
**Waterlogging**	344_WL1	BioRep1	90564764	75667990	83.55%	85.88%
	344_WL2	BioRep2	98237482	83470436	84.97%	85.28%
	344_WL3	BioRep3	95782168	82197786	85.82%	84.76%

### Differential gene expression

**Figure 3:**
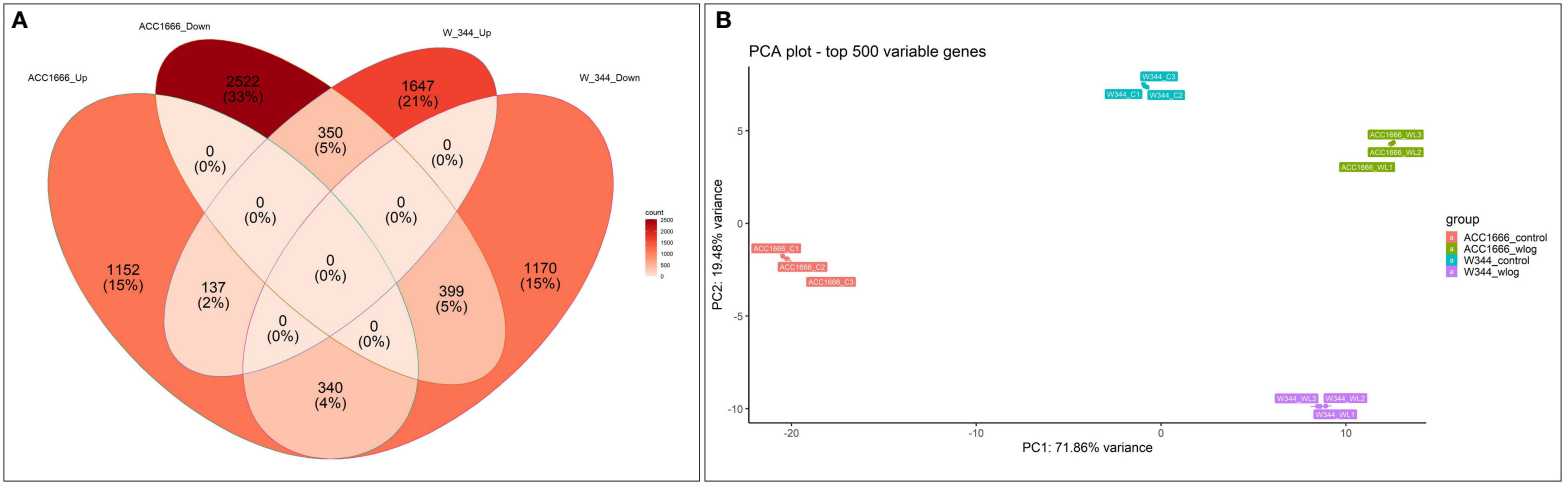
Differential gene expression pattern shown by waterlogging tolerant (Acc. 1666) and sensitive (W-344) onion genotypes under waterlogging stress using Venn diagram **(A)** and PCA **(B)**.

Differential gene expression (control *vs.* treatment) was analyzed using aligned reads of the waterlogging-tolerant (Acc. 1666) and sensitive (W-344) onion genotypes. In Acc. 1666, 1629 and 3271 genes were upregulated and downregulated, respectively, under the waterlogging condition. While in W-344, 2134 and 1909 genes were upregulated and downregulated, respectively, in response to waterlogging stress. The DEGs were further analyzed using the Venn diagram. Among the upregulated and downregulated DEGs, 137 and 399 DEGs, respectively, were common in both genotypes. By contrast, 340 DEGs were common in both genotypes but were upregulated in Acc. 1666 and downregulated in W344. Similarly, 350 DEGs were common in both genotypes but were downregulated in Acc. 1666 and upregulated in W344 (**Figure 3A**). Principal component analysis was also performed based on the top 500 most variable genes that grouped the sample with respective treatments. PC1 and PC2 explained more than 93% variation (**Figure 3B**). The top 1000 significant DEGs are schematically represented using a heatmap (**Figure S3**). Details of DEGs, such as gene count, fold change, and functional annotation, are given in **Excel S1**.

### Functional annotation of transcripts expressed in onion under waterlogging stress

**Figure 4:**
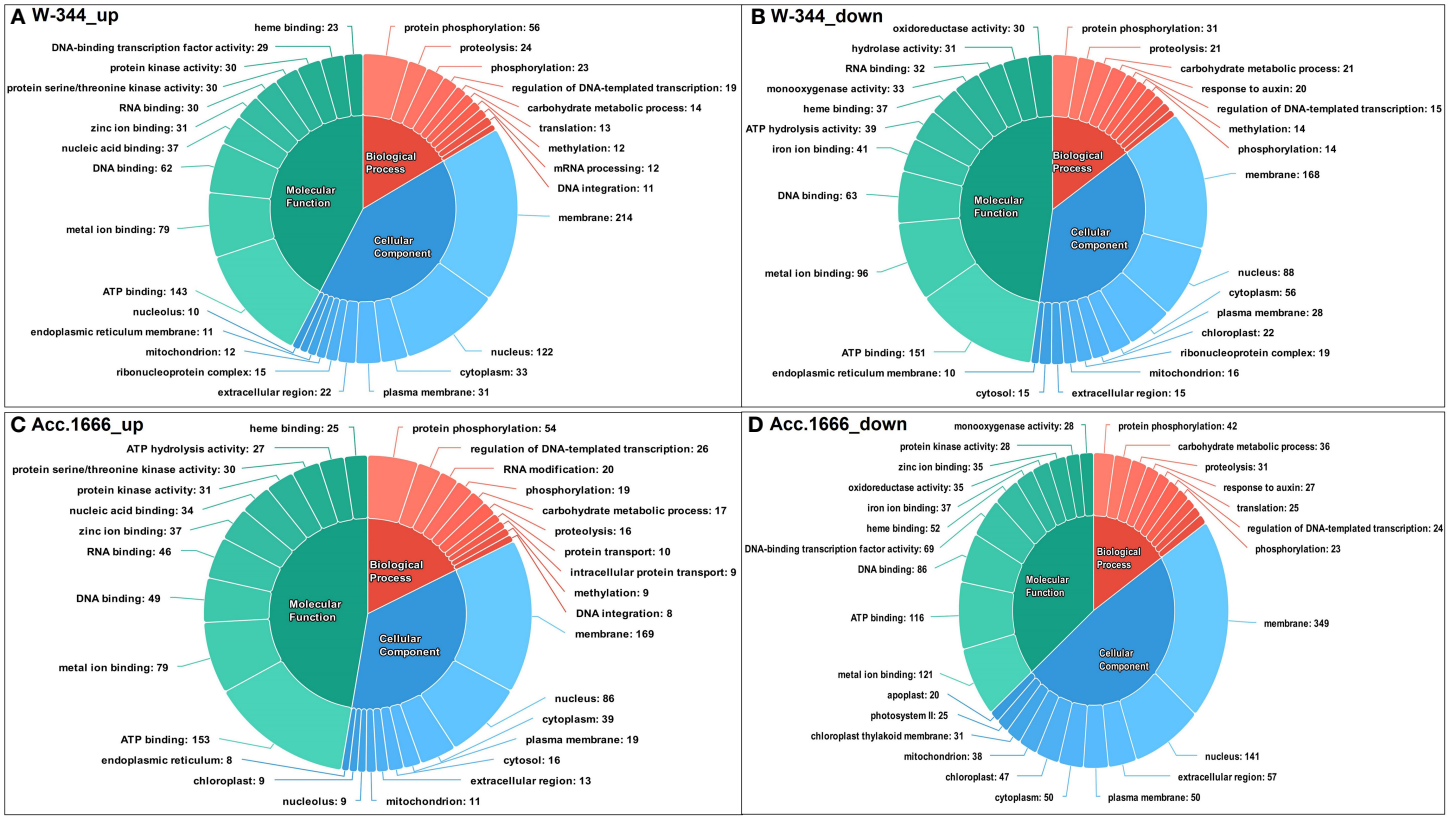
Functional annotation of differentially expressed transcripts in response to waterlogging stress in onion genotypes using Gene Ontology (GO); **(A)** Upregulated GO terms in W-344, **(B)** Downregulated GO terms in W-344, **(C)** Upregulated GO terms in Acc. 1666, **(D)** Downregulated GO terms in Acc. 1666.

The differentially expressed transcripts from both Acc. 1666 and W-344 were grouped into three categories based on the GO annotation as biological processes (BP), cellular components (CC), and molecular functions (MF). In the waterlogging-tolerant genotype Acc. 1666, GO terms such as ATP binding (153), metal ion binding (79), and DNA binding (49), which belonged to the MF category, and terms such as protein phosphorylation (54), regulation of DNA-templated transcription (26), and RNA modification (20), which belonged to the BP category, were upregulated in response to waterlogging stress. Similarly, in the waterlogging-sensitive genotype W-344, GO terms such as ATP binding (143), metal ion binding (79), and DNA binding (62), which belonged to the MF category; terms such as protein phosphorylation (56), proteolysis (24), and phosphorylation (23), which belonged to the BP category; and terms such as membrane (214), nucleus (122), and cytoplasm (33), which belonged to the CC category, were upregulated under waterlogging stress. Several DEGs in the onion genotypes contributed to GO terms involved in the plant waterlogging stress responses. The upregulated DEGs contributed majorly to GO terms belonging to the BP category, such as hydrogen peroxide catabolic process [GO:0042744], response to oxidative stress [GO:0006979], and carbohydrate metabolic process [GO:0005975], and those belonging to the MF category, such as heme binding [GO:0020037], peroxidase activity [GO:0004601], ATP binding [GO:0005524], and calmodulin binding [GO:0005516]. Downregulated DEGs majorly contributed to the GO terms such as photosynthesis, light harvesting in photosystem I [GO:0009768], fatty acid biosynthesis process [GO:0006633], abscisic acid-activated signaling pathway [GO:0009738], chlorophyll binding [GO:0016168], and heme binding [GO:0020037], which belonged to the CC category in the onion transcriptome in response to waterlogging stress. **Figure 4** presents the top 10 upregulated and downregulated GO terms in each category.

According to the analyses of distribution of clusters of orthologous groups in DEGs from Acc. 1666 and W-344, majorly belonged pathways for DNA replication and repair, post-translational modification signal transduction etc. (**Figure S4**). The transcription factor distribution was also analyzed for DEGs in contrasting onion genotypes under waterlogging stress (**Figure S5**). DEG transcripts were blasted against the plant TFDB to identify various transcription factors in the RNA-seq data. Of the identified transcription factors in both genotypes, some such as *ERF*, *bHLH*, *NFY*, *NAC*, and *bZIP* were found to be dominant in both genotypes under waterlogging stress.

### DEG expression in response to waterlogging stress

#### Phytohormone biosynthesis and signaling pathway genes

Several transcripts for phytohormone biosynthesis and signaling were differentially expressed in response to waterlogging stress in both the waterlogging-tolerant (Acc. 1666) and sensitive (W-344) genotypes (**Table 3**). Transcripts for phenylalanine ammonia lyase, a key enzyme involved in SA biosynthesis, was neutral in Acc. 1666 and downregulated by −2.4-fold in W-344. Similarly, the transcript for the enzyme involved in jasmonic acid (JA) biosynthesis, such as lipoxygenase, also exhibited differential expression in response to waterlogging stress. The transcript (g346683) coding for lipoxygenase in *Allium cepa* was upregulated by 2.3-fold in Acc. 1666 and downregulated in W344. Another gene involved in JA biosynthesis (12-oxophytodienoate reductase-1) was downregulated in both genotypes. The key gene encoding an enzyme [i.e., 9-cis-epoxycarotenoid dioxygenase (*NCED*)] involved in the ABA biosynthesis pathway was upregulated (2.3-fold) in Acc. 1666 but downregulated by 2.4-fold in W-344. One gene transcript for 1-aminocyclopropane-1-carboxylate oxidase, which is involved in ethylene biosynthesis, was upregulated. Another transcript was downregulated in Acc. 1666, whereas it was neutral in the sensitive genotype.

**Table 3:** DEGs for phytohormone in contrasting onion genotypes in response to waterlogging stress.

Transcript id	Protein name	Acc. 1666		W-344	
		Fold change	Regulation	Fold change	Regulation
g11149	Phenylalanine ammonia-lyase	–	neutral	-2.4	down
g278967	Phenylalanine ammonia-lyase	–	neutral	-2.2	down
g346683	Lipoxygenase	2.3	up	-2.3	down
g78441	Lipoxygenase	2	up	-2	down
g125937	Lipoxygenase	–	–	-4.3	down
g58476	Lipoxygenase	-2	down	–	neutral
g174553	Lipoxygenase	–	neutral	4.9	up
g373200	9-cis-epoxycarotenoid dioxygenase	2.1	up	–	neutral
g239942	9-cis-epoxycarotenoid dioxygenase	–	neutral	-2.4	down
g107775	1-aminocyclopropane-1-carboxylate oxidase	2.3	up	–	neutral

-, indicate the no fold change in the gene express, representing the neutral gene regulation.

### Energy production, carbon metabolism, and photosynthesis

Gene encoding sucrose phosphate synthase was upregulated in Acc. 1666, while that encoding sucrose synthase was upregulated in W-344. Transcripts for invertase were upregulated by 4–6 fold in both genotypes under waterlogging stress. In addition, several genes involved in the carbohydrate catabolic pathway were found to be differentially expressed in the present investigation. A key gene in photosynthesis and carbon fixation, that is, *PEP carboxylase*, was exclusively upregulated (up to 5-fold) in the tolerant genotype Acc. 1666 but downregulated in W-344 (up to 3-fold). Similarly, several photosynthesis-related genes were differentially expressed in response to waterlogging stress. The majority of the photosynthesis-related genes were downregulated and a few were upregulated in both genotypes. For example, transcripts (g539704, g9297) for the photosystem II 10-kDa polypeptide were upregulated in Acc. 1666 but downregulated in W-344. Expression of genes in anaerobic fermentation like *PDC* and *ADH* were upregulated in Acc. 1666 but their gene count was low.

### PCD, aerenchyma, and cell wall-related genes

Genes involved in PCD and aerenchyma development were differentially expressed under waterlogging stress in onion. Proteases such as ubiquitin-like protease, ubiquitin-specific protease 17, and 26S protease regulatory subunit were differentially expressed. Ubiquitin-like protease was upregulated in both onion genotypes, and 26S protease regulatory subunit was upregulated in Acc. 1666 but downregulated in W-344. Similarly, several other serine, aspartate, and cysteine peptidases were differentially expressed. Cell wall modification is among the plants’ responses to stress. In the present study, several cell wall modification-related genes were differentially expressed under the waterlogging condition such as *Expansins, Xyloglucan endotrans-glucosylase/hydrolase (XTH), Cellulase synthase, Extensin*, and *Polygalacturonases*. In Acc. 1666, nine transcripts for *Expansin* were downregulated and none was upregulated, while in W-344, four transcripts are upregulated and three were downregulated. Similarly, *XTH*, another cell wall biosynthesis-related gene, was mostly downregulated under waterlogging stress in both genotypes. Only one *XTH* gene was upregulated in Acc. 1666. One transcript for polygalacturonase was upregulated and five were downregulated in Acc. 1666, while three transcripts were upregulated and two were downregulated in W-344.

### Transcription factors

Waterlogging stress induced the expression of several transcription factors in the present study. *WRKY 70, WRKY44, WRKY22*, and *WRKY17* were upregulated by 10-, 3.8-, 2.8-, and 2.3-folds, respectively, whereas *WRKY19* was downregulated in Acc. 1666. In W-344, *WRKY12* and *WRKY40* were upregulated by 2.5- and 2.1-folds, respectively, whereas *WRKY70* was downregulated by 9.1 fold. Transcripts for the *NF-Y* transcription factor were upregulated by 4.6-fold in Acc. 1666 and downregulated by 3.2-fold in W-344. Several MYB transcription factors were differentially expressed in onion in response to waterlogging stress. Among *MYB* transcription factors, *MYB4* and *MYB29* were differentially upregulated in Acc. 1666 but downregulated in W-344. *MYB1R1* was upregulated in Acc.1666 alone. ERFs are key players in the response to various biotic and abiotic stresses in plants. ERFs in onion also exhibited differential expression under waterlogging. The majority of ERFs were downregulated in the present study. *ERF03, ERF34, ERF12, ERF39, ERF14, ERF12, ERF11*, etc. were downregulated, whereas *ERF53* and *AP2/ERF* domain-containing genes were upregulated.

### ROS-scavenging genes

Under waterlogging or hypoxia stress, antioxidant enzymes have a key role in alleviating oxidative stress induced due to ROS overproduction. Several genes encoding antioxidant enzymes were differentially regulated in onion in the present investigation. Six transcripts in Acc. 1666 and 5 in W-344 for peroxidases were upregulated, whereas 17 transcripts in Acc. 1666 and 5 in W-344 were downregulated. One transcript for CAT was upregulated in Acc. 1666, whereas none exhibited upregulation in W-344. Similarly, one transcript for CAT was downregulated in Acc. 1666, whereas three were downregulated in W-344. A transcript for APX was downregulated by 2-fold in Acc. 1666, while that for monodehydroascorbate reductase was downregulated by 2.9-fold in W-344. Other important enzymes in stress response, such as ubiquinol oxidase having alternative oxidase activity, methionine sulfoxide reductase with antioxidative activity, and respiratory burst oxidase D, were also upregulated in Acc. 1666 and downregulated in W-344. SOD was also differentially expressed in the present experiment. In both genotypes, one transcript of SOD was upregulated and the other one was downregulated. *Glutathione S-transferase (GST)* genes were differentially expressed. *GSTU1* and *GSTF1* were upregulated, whereas *GSTU3*, *GSTF2*, and *GSTF3* were downregulated in Acc. 1666. In W-344, *GSTU3* and *GSTU17* were upregulated, whereas *GSTU7, GSTF4*, and *GSTT3* were downregulated.

### Phenylalanine metabolic pathway

Genes related to the phenylalanine metabolic pathway were downregulated in the susceptible genotype (W-344), such as *PAL, 4-coumarate:CoA ligase, and caffeic acid 3-O-methyltransferase*, by 2.4, 4.6, and 2.3-folds, respectively. By contrast, *PAL* was neutral and *4-coumarate:CoA ligase* was induced by 7.9-fold in the tolerant genotype (Acc. 1666) under the waterlogging condition. Similarly, some genes involved in flavonoid biosynthesis (such as *Flavonol synthase, Flavanone 3-hydroxylase, Isoflavone 2’-hydroxylase*, and *CYP71BE30*) were also downregulated in the susceptible genotype (W-344).

### Pathogenesis-related proteins

In this study, transcripts for the defense response and pathogenesis-related proteins were also differentially expressed in response to waterlogging stress. The putative disease-resistant protein *RGA3, RGA4*, defensin, etc. were upregulated in both genotypes. By contrast, protein *TIFY* (jasmonate ZIM domain-containing protein), *MLO*-like protein, pathogenesis-related protein 10c, etc. were downregulated in both genotypes under waterlogging stress.

### Validation of DEGs under waterlogging stress through qRT-PCR

**Figure 5:**
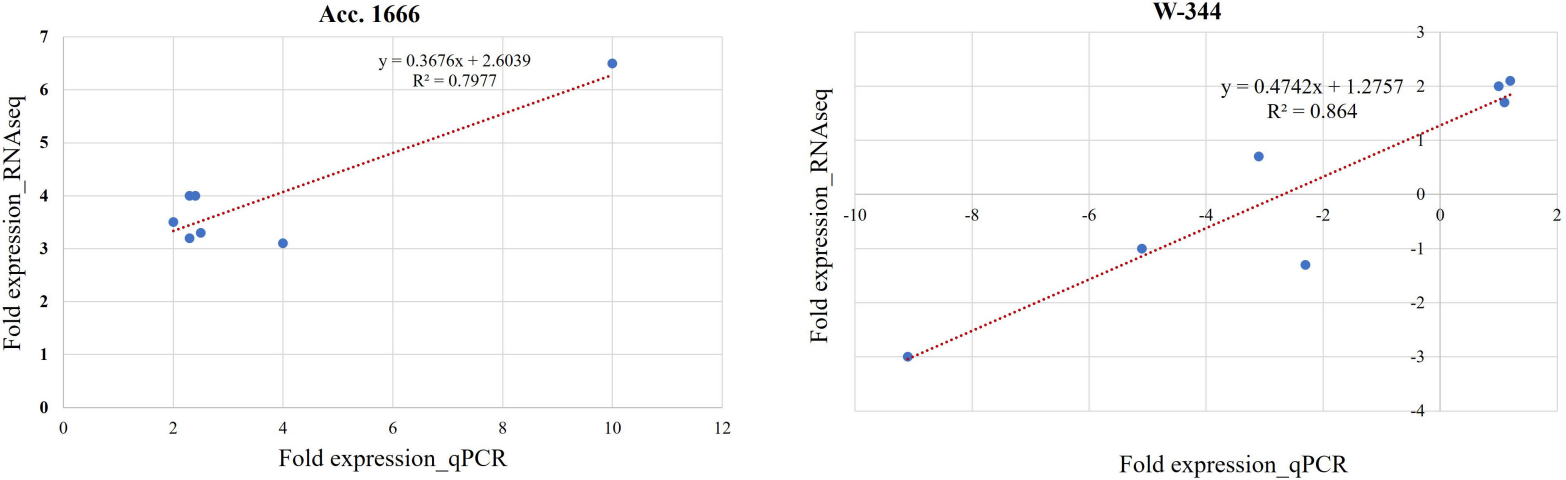
Validation of selected DEGs by qPCR analysis in Acc. 1666 and W-344. Each reaction was performed in triplicate. A good correlation was observed between RNA-Seq and qPCR data at of Acc. 1666 (R^2 =^ 79 and W-344 (R^2 =^ 0.86) after waterlogging stress in onion.

For validating the results of the transcriptome analysis through qRT-PCR, seven DEGs belonging to the categories such as antioxidant enzymes, anaerobic fermentation, phytohormones, and transcription factors and involved in waterlogging stress responses were selected randomly (**Table 1**). The gene expression level in qRT-PCR and RNA-seq exhibited a good correlation, proving the reliability of RNA-seq data (**Figure 5**).

## Discussion

### Morpho-physiological responses to waterlogging stress in onion

Onion being a shallow-rooted crop is highly sensitive to water stresses, that is, both drought and waterlogging (Pelter et al., 2004). Waterlogging is commonly caused by excess rainfall during monsoons and hinders normal plant growth and survival (Ghodke et al., 2018). Our previous study reported that during the waterlogging condition, the growth of the sensitive genotype was more severely affected than that of its tolerant counterpart. Physiological and morphological alterations were also reported in different onion genotypes in response to waterlogging stress (Gedam et al., 2022). In the present study, the physiological and molecular alterations in the leaves of the two contrasting onion genotypes (waterlogging-tolerant: Acc. 1666; waterlogging-sensitive: W-344) were investigated in response to waterlogging stress.

After 72 h of waterlogging treatment, W-344 exhibited a sensitive phenotype with reduced plant height and leaf length, whereas Acc. 1666 exhibited better plant growth with more photosynthetically active leaves, higher chlorophyll content, and good plant architecture. Leaf area and chlorophyll content are vital physiological traits severely affected under a water stress environment (Kozlowski, 1997). This may be due to a reduction in leaf cell division and elongation in the sensitive genotype. By contrast, the tolerant genotype maintained its higher leaf area and chlorophyll content under stressful environments. At the onset of waterlogging stress, photosynthesis, transpiration, and nutrient mobilization in plants are disturbed along with the degradation of photosynthetic pigments such as chlorophyll, which decreases the net photosynthesis rate (Kaur et al., 2019; Zhang et al., 2023). Consequently, the assimilate partitioning towards the leaves and developing bulbs might be hampered, accelerating premature leaf senescence. The higher photosynthesis ability, as reflected by a higher chlorophyll content in the tolerant genotype (Acc. 1666), is among the tolerance mechanisms that might help the plant to withstand the waterlogging condition. Consistently, the low chlorophyll content and poor plant phenotype with senesced leaves reflected the higher sensitivity of W-344 to waterlogging stress. The adverse effects of waterlogging stress with growth inhibition, poor plant architecture, and reduced photosynthesis and consequent low biomass and yield production have been described in sesame (Wei et al., 2013; Keya et al., 2022).

### Phytohormonal responses to waterlogging stress in onion

SA is a well-known phytohormone with a key role in various plant developmental processes including plant response to biotic and abiotic stresses. SA imparts tolerance to waterlogging stress by stimulating antioxidant enzyme activity, PCD, aerenchyma formation, adventitious root development, and improvement in the photosynthetic efficiency in various plants (Koramutla et al., 2022). Similarly, JA plays a protective role in the waterlogging condition (Kamal and Komatsu, 2016). The genes involved in JA and SA biosynthesis and signaling were more highly upregulated in the tolerant genotype (Acc. 1666) than in the sensitive counterpart (W-344). ABA is among the key plant hormones involved in plant water stress response and such as stomatal closure and aerenchyma formation. ABA promotes suberin deposition in cell wall to regulate abiotic stress responses (Shimamura et al., 2014; Zhao et al., 2021). *NCED* is a key enzyme in ABA biosynthesis, and its expression is correlated with abiotic stress tolerance in several plants (Gong et al., 2010). In the present study, *NCED* transcripts were significantly upregulated in the leaves of the waterlogging-tolerant genotype (Acc. 1666), whereas they were downregulated in the sensitive genotype. The very-long-chain fatty acids (VLFCAs) constitute the primary components of suberin in plants, and they directly participate in ethylene biosynthesis by stimulating production of 1-aminocyclopropane-1-carboxylic acid (ACC), a precursor of ethylene. The enhanced ethylene synthesis induces aerenchyma formation in root cortical cells (Yamauchi et al., 2015). Therefore, it is suggested that the ABA promoted suberin deposition in the cell wall is utilized for ethylene biosynthesis, leading to the degradation of ABA and the production of ethylene, which, in turn, induces aerenchyma formation in root cortical cells under flooding condition. Along with growth regulation, ethylene plays a major role in tolerance to abiotic and biotic stresses in plants. ACC oxidase is an ethylene biosynthesis enzyme that converts the precursor ACC to ethylene. The *ACC oxidase* gene was upregulated in the tolerant onion genotype. The gene has also been reported to be upregulated in other species such as *Arabidopsis* (Ramadoss et al., 2018), cotton (Christianson et al., 2010), and cucumber (Qi et al., 2012) in response to waterlogging stress.

### Energy production and carbon metabolism

The energy crisis is well-known in the waterlogging or hypoxia condition. Thus, modulating the expression of energy and glycolysis-related genes might help plants to overcome these adversities. The low oxygen level due to hypoxia leads to reduced ATP production (Crawford and Brändle, 1996). Under such conditions, plants need to maintain their energy level (ATP) through glycolysis and anaerobic fermentation pathways to sustain their growth and other developmental processes (Liu et al., 2020). Energy homeostasis and metabolism-related gene regulation is important in combating the adverse effect of waterlogging stress. During waterlogging, sucrose metabolism is also affected, which then hampers the source–sink relationship and ultimately leads to yield penalty (Zeng et al., 2021). In Acc. 1666, along with sucrose breakdown genes, the sucrose biosynthesis gene (SPS) was upregulated in the tolerant genotype, while all sucrose-metabolizing genes were upregulated. SPS promotes plant growth and confers abiotic stress tolerance to tomato plants (Zhang et al., 2022). In the present experiment, due to waterlogging conditions, only the roots were hypoxic, while the leaves remained in aerobic conditions. However, it is possible that some sort of signaling from the roots induced the expression of fermentation-related genes such as *PDC* and *ADH* in the leaves. Similar findings have also been reported in mungbean and cotton, where the induced expression of *PDC* and *ADH* was observed in the leaves when the roots were subjected to hypoxic conditions (Sreeratree et al., 2022; Christianson et al., 2010). Photosynthesis is highly sensitive to waterlogging stress, and several transcripts related to photosynthesis were differentially expressed in onion due to waterlogging. PEP carboxylase is among the key enzymes in C4 photosynthesis and was upregulated in Acc. 1666 but downregulated in W-344. This might have affected photosynthesis, and ultimately, carbon fixation under the stressful condition (Zhang et al., 2023). The findings thus proved that the tolerant genotype accumulates more energy by activating the alternate energy metabolism pathway under waterlogging stress, which might be responsible for its better adaptability than the sensitive genotype.

### PCD, aerenchyma, and cell wall-related genes

Plants employ several strategies to overcome prolonged waterlogging-induced oxygen deficiency. Here, the transcriptional reprogramming in leaves, and not in roots, was studied, and several genes involved in PCD and aerenchyma formation and encoding for cell wall-related enzymes were found to be differentially expressed in onion leaves in response to waterlogging stress. The study findings are consistent with those of previous studies in soybean (Chen et al., 2016), peanut (Zeng et al., 2021), and pigeon pea (Tyagi et al., 2022). In these studies, leaf tissue was used for transcriptome profiling to explore the molecular mechanism regulating the waterlogging tolerance mechanism in these crops. In addition to ROS scavenging and phytohormone biosynthesis and signaling genes, PCD-regulating genes were differentially expressed in the present study. PCD is involved in aerenchyma development under waterlogging stress in several plants (Yamauchi et al., 2018; Ni et al., 2019). In the present study, several genes, such as those encoding for ubiquitin-like protease and 26S protease regulatory subunit, and a few genes for proteases and peptidases were differentially expressed. Along with PCD, cell wall modification is crucial for aerenchyma development in roots and stems (Arora et al., 2017). Transcripts for xyloglucan-endo-trans-glucosylase/hydrolase, expansin, poly-galacturonase, etc. were differentially expressed in the onion genotype in response to waterlogging stress. Thus, this might be the factor responsible for waterlogging tolerance in Acc. 1666. Previous findings have supported these results, which indicate that anatomical adaptations such as adventitious roots and aerenchyma formation help the plants, such as rice, to withstand submergence or waterlogging (Justin and Armstrong, 1991).

### ROS scavenging and antioxidant enzyme activity under waterlogging stress in onion

Hypoxia during waterlogging leads to ROS production in plant roots as well as in leaf tissue (Baxter et al., 2014). The excessive amount of ROS is deleterious to plants, and antioxidant enzymes such as POD, CAT, SOD, and APX have a critical role in regulating ROS levels to avoid oxidative stress (Bansal and Srivastava, 2012). In the present study, significantly higher ROS (H_2_O_2_) accumulation in the sensitive genotype (W-344) indicates that this genotype experienced higher cellular membrane damage and leaf senescence. By contrast, the H_2_O_2_ level was comparatively low in the tolerant genotype, indicating less oxidative stress-induced damage and higher membrane stability and functionality under the stress condition. Consistent with low oxidative stress, the activities of various antioxidant enzymes, including SOD, CAT, POD, and APX, were significantly higher in Acc. 1666 as they might be directly involved in ROS scavenging and improving plant survival and growth under the stressful environment. In this study, the tolerance of Acc. 1666 and the sensitivity of W-344 to waterlogging stress was observed. However, waterlogging tolerance is a complex and quantitative trait and needs to be studied in detail by highlighting the adaptive mechanism regulating genes in the tolerant genotype. Information available on the molecular response of onion crops to waterlogging stress is limited. The current study was conducted to highlight the waterlogging tolerance mechanism in onion crops at the transcriptional level by using the RNA-seq approach.

The transcript level of these antioxidant enzyme-encoding genes is usually upregulated in response to abiotic stresses including waterlogging stress. Genes encoding antioxidant enzymes, such as SOD, APX, glutathione peroxidase, CAT, and GST, were differentially expressed in the present analysis. Similarly, the activities of antioxidant enzymes were also increased in response to waterlogging stress. The increased antioxidant enzyme activities under waterlogging stress have been reported in several crops such as wheat (Ma et al., 2022), onion (Dubey et al., 2020), barley (Borrego-Benjumea et al., 2020), and sesame (Sathi et al., 2022). The activities of these enzymes were higher in the tolerant genotypes of pigeon pea, maize, and sorghum (Bansal and Srivastava, 2012; Li et al., 2018; Zhang R. et al., 2019; ; Zhang Y. et al., 2019). The RNA-seq analysis revealed differential expression of antioxidant genes in tomato roots under hypoxia. These antioxidant genes were reported to be associated with aerenchyma development under waterlogging stress in tomato (Safavi-Rizi et al., 2020). In the present study, *Ubiquinol oxidase* having alternate oxidase (*AOX*) activity was upregulated in the tolerant genotype in response to waterlogging. *AOX* upregulation in hypoxia has been reported earlier (Wany et al., 2019; Jayawardhane et al., 2020). The peptide methionine sulfoxide reductase gene was upregulated in Acc. 1666. This enzyme has been reported to be involved in H_2_O_2_-induced oxidative stress response, ABA treatment, and photooxidative stress (Dai and Wang, 2010; Vieira Dos Santos et al., 2005). The respiratory burst oxidase homolog D (RbohD) gene was upregulated in the tolerant onion genotype. *AtRboh D* is involved in waterlogging response and induces the expression of ethylene biosynthesis genes as well as *ERFs*, *ADH1*, and *PDC1* under the waterlogging condition (Yang and Hong, 2015; Liu et al., 2017). Thus, the RbohD might be involved in plant response to waterlogging stress.

A higher transcript level and more enzyme activities during waterlogging indicate a translational correlation between gene expression and protein-level actions. In the present study, some transcripts for a particular enzyme were upregulated, whereas some were downregulated. However, the enzyme activity was higher under the stress condition. Glanemann et al. (2003) also found a similar disparity between the mRNA abundance and enzyme activity. They stated that predicting enzyme activity from transcriptome data is difficult. The variation in the transcript level at different time points and in tissue types, and resources of translation significantly influence this correlation. Thus, in many scenarios, determining only the mRNA level is not sufficient for predicting the gene product (protein) level. Alternative splicing and post-transcriptional modifications also affect the mRNA–protein correlation (Brar et al., 2012; Liu et al., 2016). This report thus suggests that the high antioxidant enzyme activity confers stress tolerance to the tolerant onion genotype Acc. 1666.

### Transcription factor expression under waterlogging stress

Transcription factors act as key regulators of the waterlogging stress response (Valliyodan et al., 2014). In the present study, several transcription factors were differentially expressed in response to waterlogging stress. Members of transcription factor families such as *ERF, WRKY, MYB, NAC*, and *bHLH* are known to be involved in regulating various biotic and abiotic stress responses in onion (Ghodke et al., 2020; Khandagale et al., 2022). These transcription factors are also involved in response to a hypoxic condition caused by waterlogging stress in plants. *WRKY22* was involved in submergence tolerance by positively regulating ethylene-mediated signaling (Hsu et al., 2013). *WRKY40* has also been reported to be upregulated under waterlogging stress in *Arabidopsis* and Kiwifruit (Meng et al., 2020; Wurms et al., 2023). *WRKY70* is known to regulate SA and JA signaling in response to stress and acts as a negative regulator of senescence (Besseau et al., 2012). *NF-Y* regulates ABA synthesis by binding to the *NCED* gene promoter (Bi et al., 2017). In addition to ABA regulation, *NF-Y* improves photosynthesis, activity of antioxidant enzymes (Manimaran et al., 2017), and root length (Han et al., 2013). MYB transcription factors are crucial players in anthocyanin and phenylpropanoid biosynthesis and plant resistance to biotic and abiotic stress. *MYB1R1* expression was induced in hot pepper under waterlogging stress (Zhang R. et al., 2019; Zhang Y. et al., 2019). Further, *MYBs* are involved in response to the hypoxia and waterlogging stresses (Lee et al., 2007; Fukushima et al., 2016). Ethylene is a gaseous hormone having a vital role in controlling plants’ waterlogging responses (Khan et al., 2020). The *ERF* family is a plant-specific transcription factor group regulating plant responses to several abiotic stresses (Muller and Munne-Bosch, 2015). The differential expression of these transcription factors indicates their importance in transcriptional reprogramming during adaptation to the waterlogging condition.

### Phenylalanine metabolic pathway

Lignin biosynthesis genes, such as *4-coumarate:CoA ligase* and *caffeic acid 3-O-methyltransferase*, were associated with the waterlogging response in wheat (Nguyen et al., 2016). *Arabidopsis* mutants for these genes accumulated a low amount of lignin than the wild-type plants (Vanholme et al., 2012). Induced expression of *4-coumarate:CoA ligase* was also reported in *Sesbania cannabina* under waterlogging stress (Ren et al., 2017). Similarly, flavonoid biosynthesis-related genes were downregulated in the susceptible onion genotype, whereas these were neutral in the tolerant genotype. RNA-Seq of *Pterocarya stenoptera* and *Brassica napus* leaves revealed that flavonoid synthesis-related genes were associated with the waterlogging stress response (Li et al., 2021; Hong et al., 2023). Thus, upregulation of these genes of the phenylalanine metabolic pathway in the tolerant onion genotype might play some role in conferring waterlogging stress tolerance.

## Conclusions

In this study, two contrasting onion genotypes with varying degrees of waterlogging tolerance capacities were subjected to 72 h of waterlogging stress. Morphological and physiological stress indicators revealed that Acc. 1666 exhibited a stronger tolerance to waterlogging than the sensitive genotype W-344. Genes coding for proteins regulating several key biological processes involved in combating waterlogging stress, such as phytohormone production, antioxidant enzymes, PCD, anaerobic fermentation, and energy production, were differentially expressed. Genes such as *ADH, PDC, WRKY22*, and *RbohD* were upregulated in the tolerant genotype and might have been involved in conferring stress tolerance to Acc. 1666. This study provides valuable information about key regulatory genes that can be used in onion breeding programmes aimed at developing waterlogging-tolerant varieties.

## Data availability statement

The data from the current study has been made available publically at NCBI with accession no “PRJNA926808”.

## Author contributions

PG, AT, SG, AK: conceptualization, planning, methodology, supervision, writing-original draft., project administration. KK, DS, KB, YK: methodology, biochemical analysis, statistical analysis. VM, AG: resources. OK, VB: methodology, writing, editing. MS: supervision, funding, and project administration. All authors listed have made a substantial, direct, and intellectual contribution to the work.
