# Supplementary material for: Elucidating the molecular responses to waterlogging stress in onion (Allium cepa L.) leaf by comparative transcriptome profiling
Source: Front Plant Sci. 2023 Aug 8;14:1150909. doi: 10.3389/fpls.2023.1150909 (PMC10442827; doi:10.3389/fpls.2023.1150909)
Supplement: Supplementary file 1 [file DataSheet_1.docx]

**Supplementary material**

**Elucidating molecular responses to waterlogging stress in onion (*Allium cepa* L.) leaf by comparative transcriptome profiling**

Pranjali Gedam^1^, Kiran Khandagale^1^, Dhananjay Shirsat^1^, A Thangasamy^1^, Onkar Kulkarni^3^, Abhijeet Kulkarni^3^, Swaranjali S. Patil^3^, Vitthal T. Barvkar^2^, Vijay. Mahajan^1^, A. J. Gupta^1^, Kiran P. Bhagat^4^; Yogesh P. Khade^1^, Major Singh^1^, Suresh Gawande^1*^

^1^ICAR-Directorate of Onion and Garlic research, Pune, India, 410505

^2^Department of Botany, Savitribai Phule Pune University, Pune, India, 401007

^3^Bioinformatics Centre, Savitribai Phule Pune University, Pune, India, 401007

^4^ICAR-Directorate of Floriculture Research, Pune, India, 411005

*Corresponding author: sureshgawande76@gmail.com

**1 Supplementary Data**

Supplementary Excel S1. All differentially expressed genes in both genotypes along with their functional annotation in response to waterlogging stress

**2. Supplementary Figures and Tables**


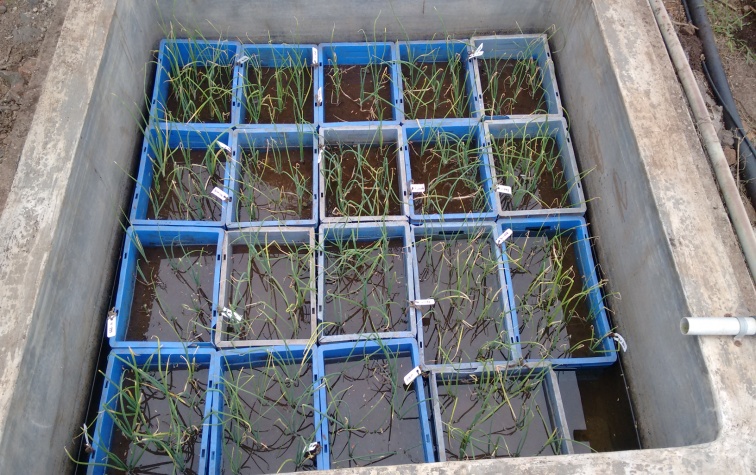


**Supplementary Figure S1:** Experimental setup for waterlogging stress

**
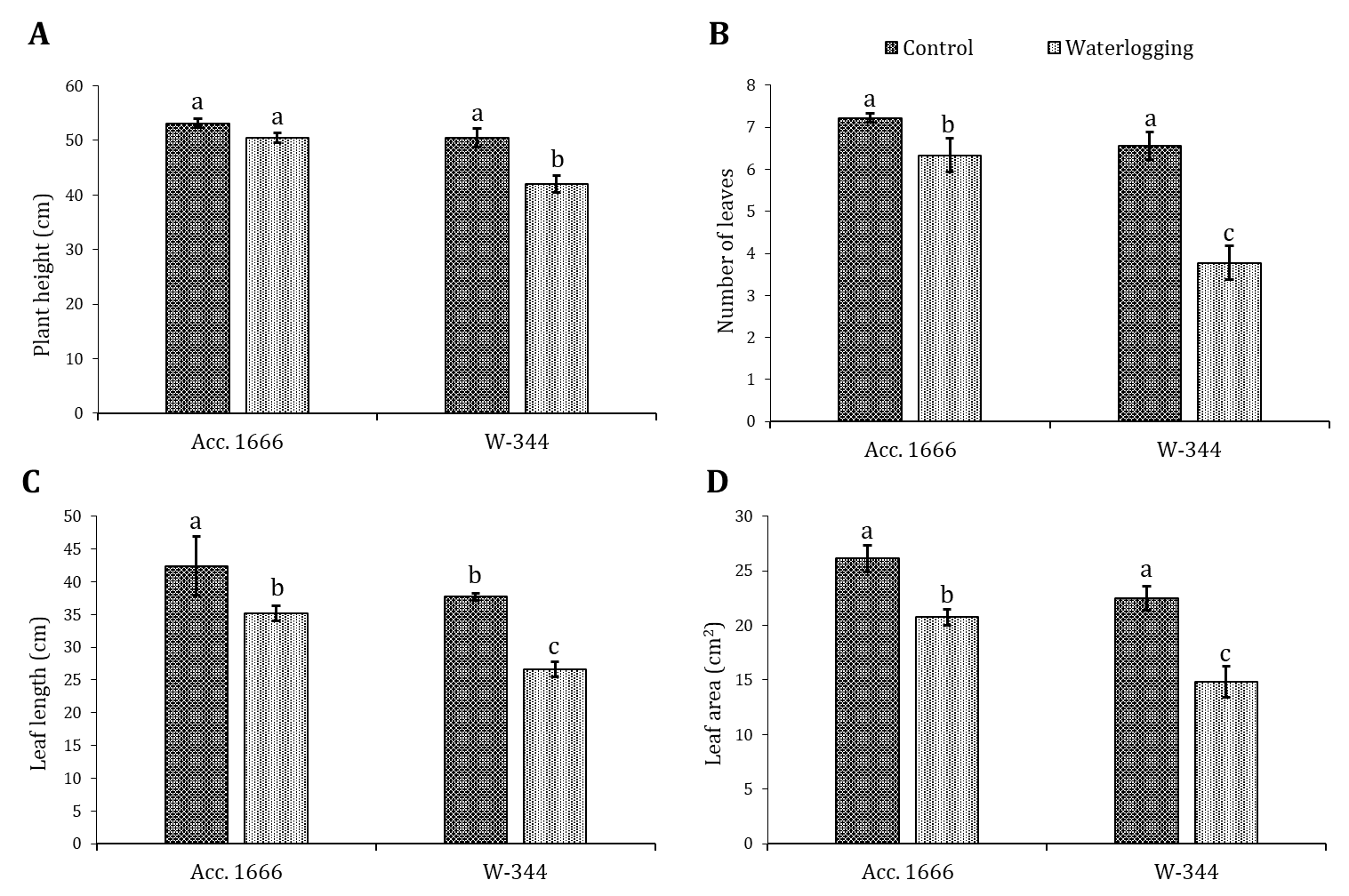
**

**Supplementary Figure S2:** Morphological parameters under the waterlogging stress in contrasting onion genotypes

**
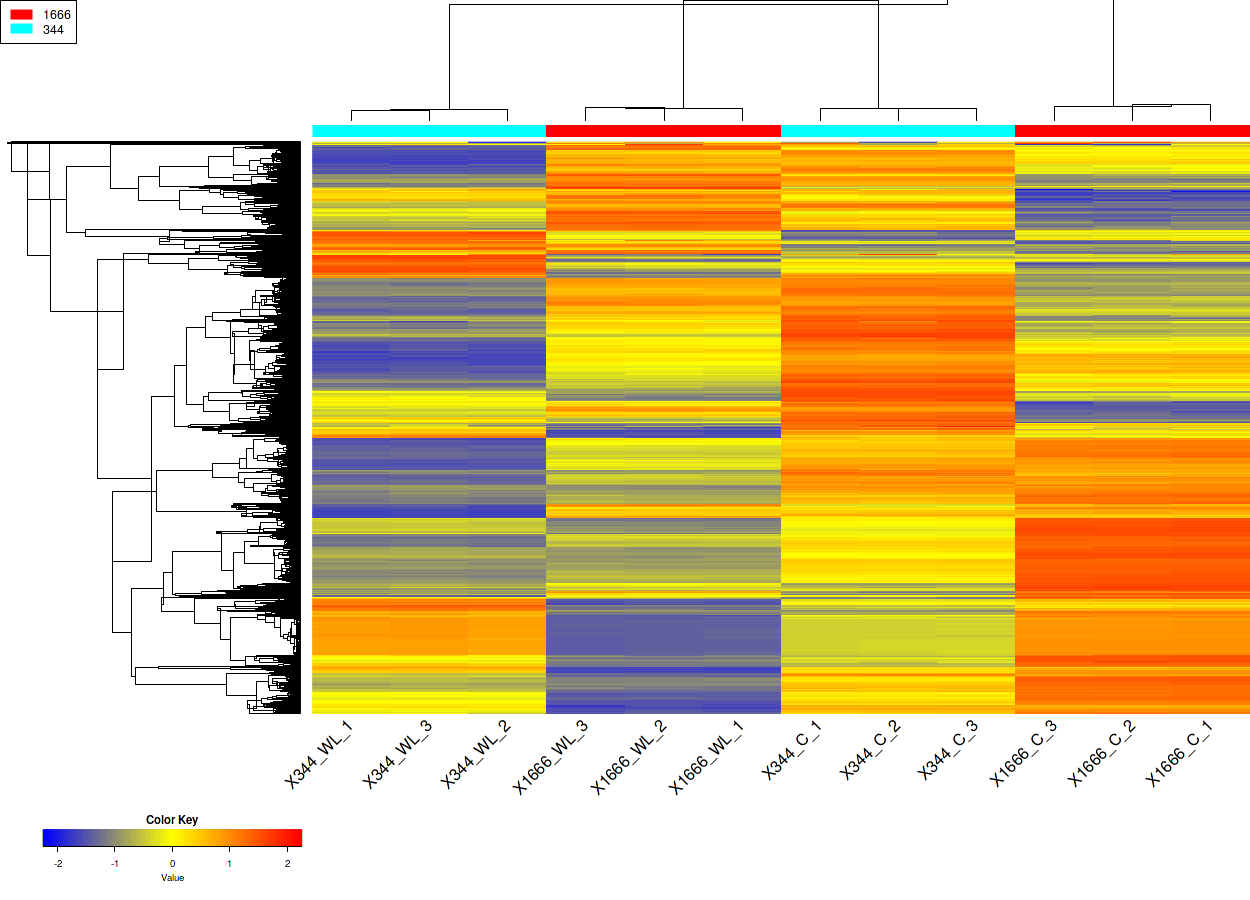
**

**Supplementary Figure S3.** Differential expression pattern of top 1000 most variable DEGs in onion genotypes in response to waterlogging stress. Normalised gene count values are used for this heat map preparation.


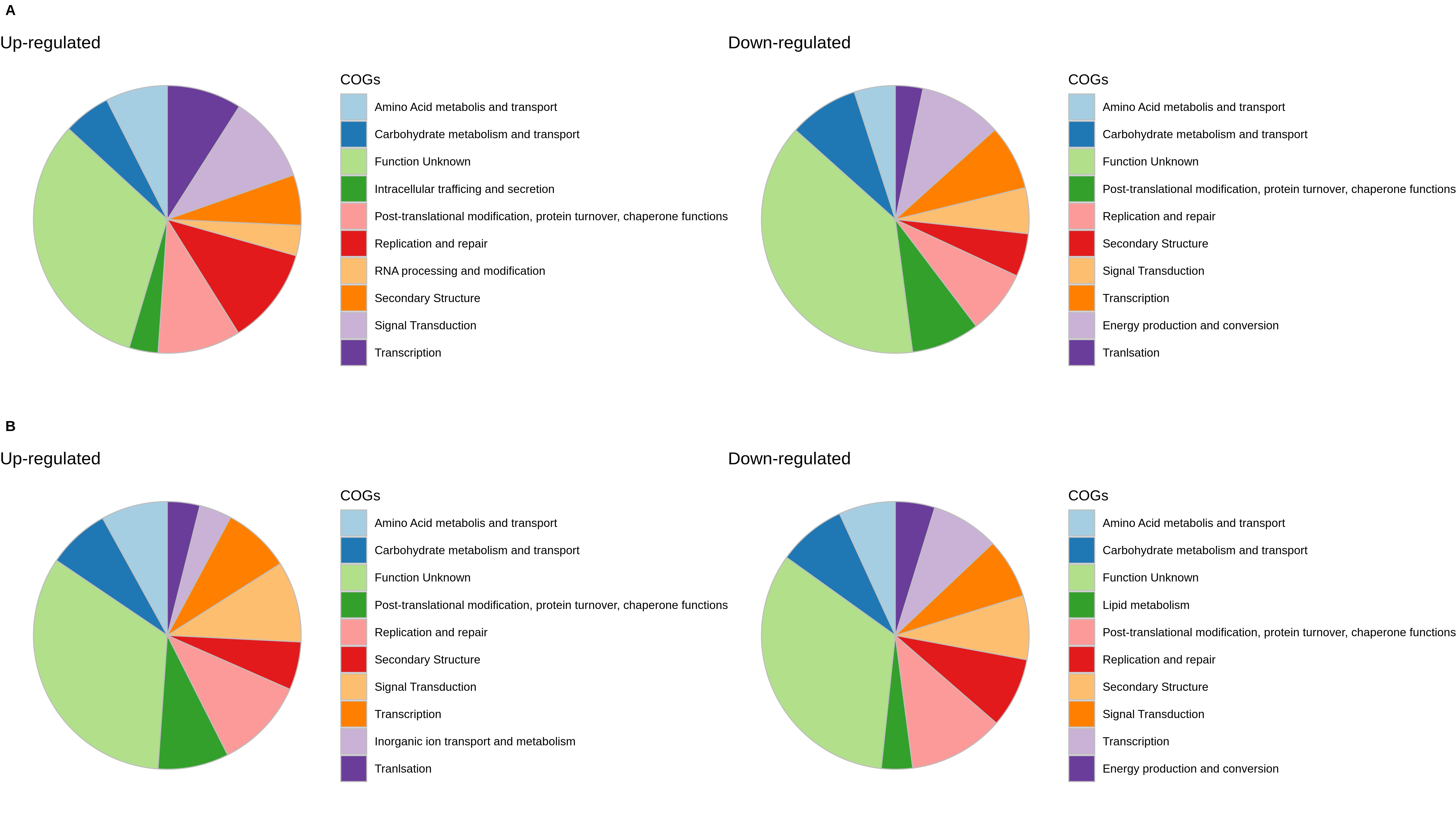


**Supplementary Figure S4:** Functional annotation of differentially expressed transcripts in response to waterlogging stress in onion genotypes using COG categories; A. Acc. 1666, B. W-344


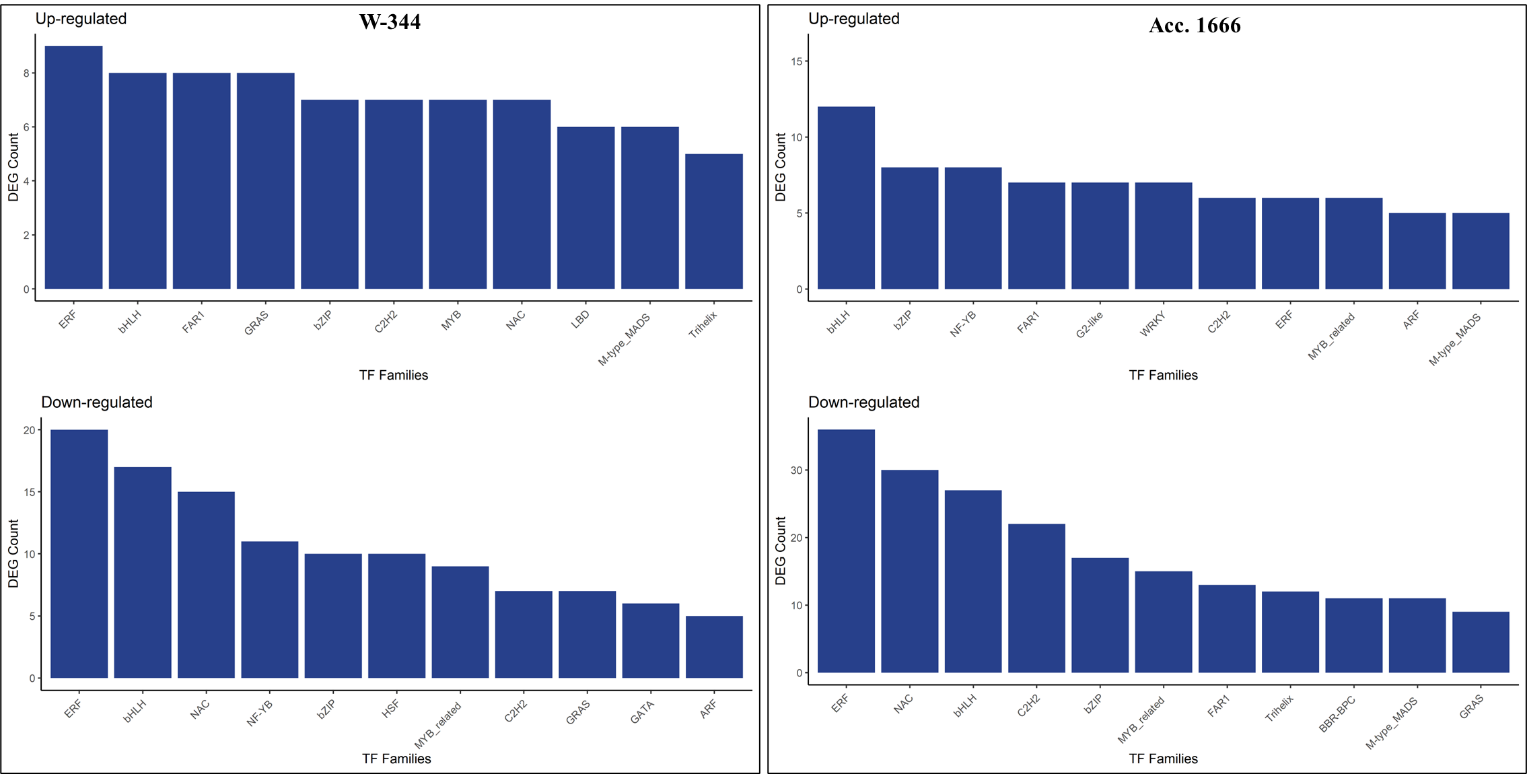


**Supplementary Figure S5.** Transcription factor distribution of transcripts expressed in onion genotypes in response to waterlogging stress; X axis denotes TF families and Y axis represents DEG count for each TF family in the dataset.
